# Emerging Research Topics in the Vibrionaceae and the Squid–*Vibrio* Symbiosis

**DOI:** 10.3390/microorganisms10101946

**Published:** 2022-09-30

**Authors:** William Soto

**Affiliations:** Integrated Science Center Rm 3035, Department of Biology, College of William & Mary, 540 Landrum Dr., Williamsburg, VA 23185, USA; wsoto@wm.edu

**Keywords:** Vibrionaceae, host-microbe interactions, symbiosis, squid–*Vibrio* mutualism, bioluminescence

## Abstract

The Vibrionaceae encompasses a cosmopolitan group that is mostly aquatic and possesses tremendous metabolic and genetic diversity. Given the importance of this taxon, it deserves continued and deeper research in a multitude of areas. This review outlines emerging topics of interest within the Vibrionaceae. Moreover, previously understudied research areas are highlighted that merit further exploration, including affiliations with marine plants (seagrasses), microbial predators, intracellular niches, and resistance to heavy metal toxicity. Agarases, phototrophy, phage shock protein response, and microbial experimental evolution are also fields discussed. The squid–*Vibrio* symbiosis is a stellar model system, which can be a useful guiding light on deeper expeditions and voyages traversing these “seas of interest”. Where appropriate, the squid–*Vibrio* mutualism is mentioned in how it has or could facilitate the illumination of these various subjects. Additional research is warranted on the topics specified herein, since they have critical relevance for biomedical science, pharmaceuticals, and health care. There are also practical applications in agriculture, zymology, food science, and culinary use. The tractability of microbial experimental evolution is explained. Examples are given of how microbial selection studies can be used to examine the roles of chance, contingency, and determinism (natural selection) in shaping Earth’s natural history.

## 1. Introduction

### 1.1. The Vibrionaceae

The bacterial family Vibrionaceae (Class Gammaproteobacteria) encompasses a cosmopolitan group of Gram-negative rods, straight or curved, which are mostly aquatic and possess tremendous metabolic and genetic diversity [1]. The family contains two circular chromosomes, one large and the other small (both circular). Vibrionaceae isolates can sometimes be found in terrestrial habitats, when aquatic habitats are nearby. Vibrionaceae populations residing in such ecotones may be subjected to source-sink dynamics [2]. The family is mostly motile with at least one polar flagellum but more are possible. However, the gut symbiont *Vibrio halioticoli* to the abalone *Haliotis discus hannai* is non-motile [3]. Cellular dimensions are typically 1 μm in width and 2–3 μm in length, with most species oxidase positive. Oxidase-negative species include *Vibrio aerogenes*, *Vibrio gazogenes*, and *Vibrio metschnikovii* [4]. The family is facultatively anaerobic, having respiratory (aerobic and anaerobic) and fermentative metabolisms [1]. The two most speciose genera are *Vibrio* and *Photobacterium*. Bioluminescence has long been recognized in these two genera but has more recently been documented in *Enterovibrio* and *Photodesmus* [5,6]. Vibrionaceae members can exist as free-living bacterioplankton or engage in host-microbe interactions as pathogens, commensals, or mutualists [7]. Their hosts can be multicellular organisms and single-celled eukaryotic microbes [8]. In metazoans, Vibrionaceae can be part of the native gut microbiota [9]. Additionally, Vibrionaceae can adopt a biofilm lifestyle in sediment or by attaching to suspended particulate matter (colloids), marine snow, detritus, and floating debris [7]. Of course, biofilms are possible on the surfaces of host organisms as well. The Vibrionaceae includes species that are pathogenic to aquatic animals and humans, including *Vibrio cholerae*, *Vibrio parahaemolyticus*, and *Vibrio vulnificus* [1]. *Vibrio harveyi* and *Vibrio anguillarum* cause considerable economic losses to the aquaculture industry worldwide [10]. Verily, there is a growing interest in using benevolent Vibrionaceae as probiotics to combat confamilial pathogens in aquaculture to “fight fire with fire” [9]. This approach has led to multiple successful case studies in aquaculture, counting scallops and salmon [11]. In one study, an innocuous strain of *Vibrio alginolyticus* was able to provide some protection to fish from the pathogens *Vibrio anguillarum* and *Vibrio ordalii*. Given the importance of the Vibrionaceae, this taxonomic group merits continued and deeper research in a multitude of areas. This review outlines and explains emerging topics of interest due to investigating the Vibrionaceae. Moreover, subjects worthy of additional scientific inquiry are likewise detailed for realms priorly understudied. The squid–*Vibrio* symbiosis is a stellar model system, which can be useful in these undertakings. Where appropriate, the squid–*Vibrio* mutualism is mentioned in how it has or could facilitate the illumination of these various subjects.

### 1.2. The Squid–Vibrio Symbiosis

**Figure 1:**
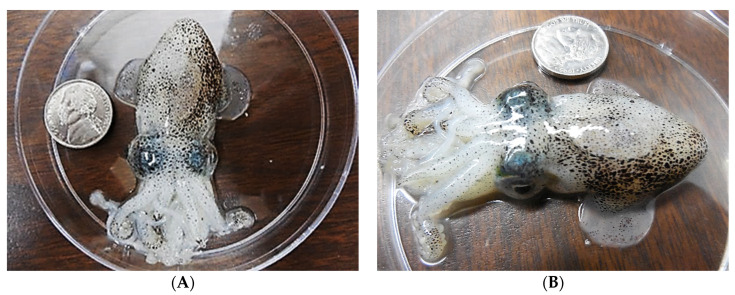
(**A**,**B**) show an adult six-month old *Euprymna* female squid from a top and side view, respectively. In captivity, adults can live up to about ten months. Females become reproductively active within 3 months of hatching. An American 5-cent coin (5¢, a nickel, diameter = 21.2 mm) provides scale. Pictures were taken with an HD Nikon L610 digital camera.

**Figure 2:**
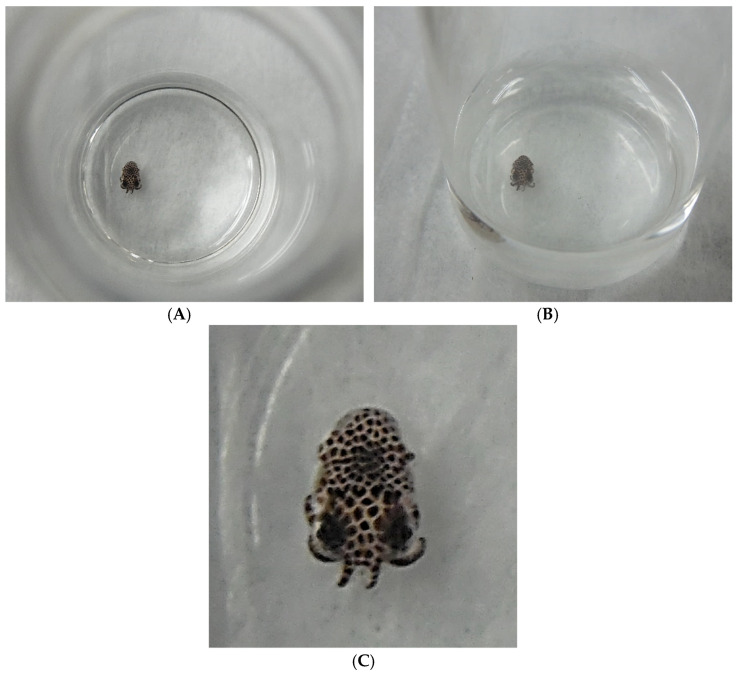
(**A**) (top view), (**B**) (oblique or slanted view), and (**C**) show a *Euprymna* squid hatchling a few hours after egg emergence. The hatchling is in a 10-mL scintillation vial with 5.0 mL 34 ppt artificial seawater (Instant Ocean). (**A**,**B**) are meant to provide scale as to the actual size of a typical “newborn” squid hatchling. The diameter of the 10-mL scintillation vial is nearly identical to an American 25-cent coin (25¢, a quarter, diameter = 24.3 mm). (**C**) is a close-up of the animal for easier viewing of the chromatophores. Pictures were taken with an HD Nikon L610 digital camera.

The symbiosis between the marine bioluminescent bacterium *Vibrio fischeri* and sepiolid squids (Figure 1) in the genus *Euprymna* is an established model system for investigating associations between bacteria and animals [12]. This symbiosis has not only been useful in illuminating research questions in mutualisms, but it has also been helpful and valuable in elucidating puzzles in commensalisms and parasitisms [10]. *Vibrio fischeri* has close relatives that cause infectious diseases in animals and humans, counting *Vibrio cholerae*, the causative agent of cholerae. The squid inhabit shallow sandflats along coastal ranges throughout regions in the Indo-West Pacific. Within the squid, the bacteria reside in a morphologically complex, specialized structure called the light organ, a nutrient-rich microenvironment relative to the free-living surroundings (water column, sediment, etc.) outside the animal. As a result, the symbionts receive an ample food supply. The squid are active at night (nocturnal), a time when they hunt and forage for food, establish territorial ranges, and search for mates to reproduce [10]. Sepiolid squids use the bioluminescence produced by the bacterial symbionts for counterillumination, which enables stealth and covert roaming at nighttime amid diffuse but bright light that stems from celestial sources (moon and stars), nightglow, auroras, zodiacal light, and gegenschein [13]. Accordingly, counterillumination empowers the squid with disguise and concealment in down-welling light, as there is less contrast between the animal’s silhouette and the surrounding environment. During the day, the squid sleep and remain buried in the sand. Within a few hours after first emerging from their eggs at dusk, axenic squid hatchlings (Figure 2) are infected by free-living *Vibrio fischeri* from the oceanic water column, the sediment, or egg external surface. Consequently, the bacterial symbionts are transmitted horizontally (not vertically) from one host generation to the next [14]. Sometimes the term “environmental” transmission is used instead of “horizontal”. Hatchling squid are colonized over the course of minutes to hours, during which the founding bacterial cells navigate the light organ tissues and ultimately access the gnotobiotic luminal spaces of the deep crypts. In the crypts, the bacteria that successfully colonize undergo exponential growth (with a 20–30 min generation time) and reach a population plateau before dawn. At dawn, 90% to 95% of the bacterial light organ population is vented (expelled) by the squid host to the outside marine environment. The remnant population in the squid light organ once again undergoes exponential growth and plateaus to pre-expulsion levels by nightfall [15].

## 2. Host-Microbe Interactions with Marine Plants

Only a handful of Vibrionaceae species can persist in freshwater habitats for extended periods, like *Vibrio qinghaiensis*, *Vibrio cholerae*, *Vibrio mimicus*, and *Vibrio parahaemolyticus* [13,16,17]. *Photobacterium damselae* has been isolated from freshwater [18], and *Photobacterium phosphoreum* can dwell on the outer surfaces of salmon swimming in freshwater [19]. Through much of its natural history, the Vibrionaceae has largely been a family of marine microorganisms [13]. Furthermore, the Vibrionaceae is repute for its ability to engage in host-microbe interactions with eukaryotic (single-celled and multicellular) organisms, including pathogenic, commensal, and mutualistic associations [10]. Despite these facts, the totality of relationships the Vibrionaceae forms with marine plants (i.e., seagrasses) is a sphere that has received insufficient attention. [For the purposes of this review, algae will not be considered as true plants.] In some sense, the host-microbe interactions that the Vibrionaceae initiates with brackish and marine plants are chiefly uncharted waters, relative to other subjects that have been examined in association with this bacterial family. Much in this field remains an enigma yet to be unraveled. Indeed, in some instances published reports even seem to provide conflicting conclusions. Some temperate *Zostera marina* seagrass beds are believed to lower the abundance of the Vibrionaceae in the leaf canopy [20]. However, other studies imply the Vibrionaceae (including bioluminescent isolates and animal pathogens) may actually be a native and prolific member of the epiphytic microbial community that inhabits seagrass meadows, including the leaves, rhizomes, and roots [21]. Vibrionaceae has also been isolated from the sediment of seagrass meadows, including nitrogen-fixing species like *Vibrio diazotrophicus* [22]. Undoubtedly, the Vibrionaceae can be highly abundant in seagrass beds [23,24].

These observations alone raise several captivating questions. Are there nitrogen-fixing symbioses between marine plants and the Vibrionaceae? If so, do nodule-like structures form in the seagrass rhizosphere as with leguminous plants with *Rhizobium*? How does the Vibrionaceae protect its nitrogenases against oxygen toxicity? Why are there bioluminescent and pathogenic Vibrionaceae (e.g., *Vibrio alginolyticus* and *Vibrio anguillarum*) associated with marine plants [25,26]? Are there intimate bioluminescent symbioses between Vibrionaceae and marine plants that have gone undetected by researchers? If so, do marine plants house the bioluminescent bacteria in a morphologically specialized light organ? Are there Vibrionaceae isolates or strains that are pathogenic to seagrasses? Are there complex interactions between the Vibrionaceae and seagrass host immunity? Can seagrass meadows serve as reservoirs for Vibrionaceae members that are pathogenic to animals? Understanding host-microbe relationships and the infectious diseases of aquatic plants is especially important within the context of climate change [27,28]. In fact, there is great interest in using the host-microbe associations of marine plants as “indicator superorganisms” for measuring environmental health and understanding ecosystem function [29,30,31]. In part, this is because seagrasses are ecologically sensitive species, since they have the highest light requirements of all angiosperms. Correspondingly, seagrasses are quite sensitive to environmental conditions that change water clarity and quality, like eutrophication, pH fluxes, colloid accretion, and continual sediment disturbances (trawling) causing silt to remain suspended in water [32]. For this reason, the “holobiont” or “hologenome” concept has been advanced for the studying of host-microbe interactions with marine plants [33], yet this proposal has not been without criticism [34].

The affiliations that materialize between marine plants and microorganisms also have industrial applications for the discovery of useful bioactive compounds, including in medicine and nutraceuticals [35]. There has also been a growing interest in developing marine plants (*Syringodium filiforme* and *Enhalus acoroides*) as a new potential food source for human consumption or culinary use [36,37]. Edible seagrasses offer a practical vegetable alternative for coastal communities, especially in developing countries, where arable land might be in short supply. Coastal areas might have sandy soils with low fertility, low freshwater availability, high evaporation rates, and high salinity due to wind-blown salt and saltwater intrusion. The nutrient content of edible marine plants is comparable to typical terrestrial crops, like rice, wheat, and cassava [36,37]. In some cases, the nutrition from marine plants can be genuinely better, including higher fiber and mineral content (Ca, P, and Fe). There is also interest, especially in Asia, in using marine plants (*Zostera marina*) for the development of novel alcoholic beverages and special fermented food sauces akin to soy sauce or doubanjiang [38,39]. There is an emerging enthusiasm by entrepreneurs to create a new marine fermentation industry, and much food science research and funding is currently being invested toward this venture. Another potential application of marine plant (*Cymodocea serrulata* and *Thalassia hemprichii*) fermentation is the production of biofuels like ethanol and hydrogen gas [40,41]. *Vibrio aerogenes* may be useful here, since it produces H_2_ during fermentation [4]. Assuredly, these research and development efforts in fermented marine foodstuffs, beverages, and other commodities have already been demonstrated with algae with promising success, and seagrasses are now being seen as the next frontier [42]. Food science research, zymology, agriculture, and culinary science in Asia have had extensive experience and success in obtaining economically useful and valuable products from algae and aquatic plants [43,44]. This progress has helped drive immense interest in biorefineries [45].

If seagrasses are going to be pursued as a plant crop for economic interests at a large industrial scale, or even just small niche markets, then understanding their host-microbe associations will be imperative to establish efficient farming and cultivation practices that will maximize the commercial value of the derived products [39]. Here, knowledge of the Vibrionaceae could be helpful, as a species might be identified that could increase crop yield or plant hardiness. Alternatively, a Vibrionaceae isolate might be ascertained to be antagonistic to a well known seagrass pathogen (*Labyrinthula*), which could then serve as a biocontrol agent in seagrass agriculture [27,33]. Since there are Vibrionaceae species that produce cellulases (e.g., *Vibrio xiamenensis* and *Photobacterium panuliri*), seagrass phytopathogens are conceivable [46,47]. Seagrass meadows are known to produce antimicrobial and antibiofilm compounds, including against Vibrionaceae [48]. Extracts from *Halophila ovalis* have shown antioxidant and anti-inflammatory activities [49], while *Posidonia oceanica* displayed anti-protistal effects against *Trypanosoma* and *Leishmania* [50]. Impressively, extracts from marine plants (e.g., *Halodule pinifolia*) can even have larvicidal effects against mosquitoes that are disease vectors [51]. Seagrasses are also known to have anti-cancer, antifungal, antiviral, and numerous other properties. An important consideration here is that seagrass microbial symbionts can influence the bioactive substances that plants produce. Furthermore, the seagrass microbiome is also known to produce antimicrobials against other microorganisms that are nonnative to the plant host [52]. For example, the residential microbiota of a seagrass bed can produce substances that disrupt quorum sensing (quorum quenching) in foreign microbes, which impedes biofilm formation and prevents attachment to the plant host by the newcomers [53,54]. Microbial mutualists (autochthonous) may be responsible for protecting the seeds of seagrasses against phytopathogens or other uninvited microorganisms (allochthonous). Thus, indigenous microbial symbionts help increase seed survival and germination [52]. Aptly, marine plants and their microbiomes, which would include the Vibrionaceae, are an underexplored treasure trove for the bioprospecting of antimicrobial compounds and other bioactive molecules [24,51]. Although the focus here has been on seagrasses, host-microbe interactions that the Vibrionaceae forms with other aquatic plants should not be overlooked, including salt-tolerant freshwater plants (the halophyte *Ruppia maritima*) and mangroves [55]. Since there are a few Vibrionaceae members that can persist at low salinities, the relationships this taxonomic family engages in with freshwater plants should also receive further rigorous treatment. Generally, host-microbe relationships between the Vibrionaceae and aquatic plants have been severely understudied.

## 3. Microbial Predators, Facilitation of Virulence, and Coincidental Evolution Hypothesis

In the environment, viruses and protists are known to be major predators of prokaryotes, including the Vibrionaceae [56]. Hence, from one view point, viruses and protists are noteworthy in their potential to check pathogen populations in nature. However, another perspective is also warranted for these microbial “beasts of prey”. Although viruses and protists consume tremendous prokaryotic biomass each year, they also conceivably enable virulence in bacteria. Phages are well recognized by scientists in their ability to bestow virulence factors to their prey, including the cholera toxin and the toxin coregulated pilus in *Vibrio cholerae* [57,58]. Yet, a more recent finding has been that viruses and protists can facilitate the bacterial acquisition of virulence factors through coevolution with their prey (Red Queen hypothesis) [59]. To alleviate viral parasitism and protist predation, bacteria have been under positive selection pressure to procure certain adaptive traits. The acquisition of these adaptive phenotypes, as part of an ancient adversarial relationship with phages and protists, is what has actually engendered the evolution of bacterial pathogens [56,60]. Thus, the rise of bacterial pathogens is mostly not due to an evolutionary association or an arms race that has existed with multicellular hosts. This supposition has been termed the “coincidental evolution” hypothesis [61,62]. For example, the Type III secretion system (T3SS) delivers virulence effector proteins from many bacterial pathogens to eukaryotic host cells. Nonetheless, the T3SS originated in bacteria hundreds of millions of years before major lineages of multicellular hosts first appeared. Many bacteria that are able to resist grazing or digestion by protists possess a T3SS [61]. Additionally, phages promote horizontal gene transfer and genomic rearrangements [63], including the movement of pathogenicity islands [64]. This is especially relevant with the Vibrionaceae, since this taxonomic family has specialized genomes that contain integrons which can capture mobile genetic elements, replicons, or gene cassettes [65]. Parasitic DNA, like retrotransposons and homing endonucleases, can also play a similar role [66,67].

Viruses themselves can protect bacteria from predatory protists; lysogens carrying prophages and mobile genetic elements can encode cytotoxins that kill grazing protists. Viruses may adopt a latent infection or lysogenic strategy when bacterial hosts are not abundant, thus ensuring a continuous chain of host transmission; an extirpated or extinct bacterial host can be an evolutionary dead end for a virus [56]. These same cytotoxins can kill phagocytic immune cells in multicellular hosts. When such cytotoxins kill bacterivorous protists, the protists themselves become food for the bacteria, which can lead to large population blooms. Ironically, the hunter becomes the prey. Such bacterial population blooms can also benefit the latent viruses or prophages by increasing the quantity of their permissive hosts [56]. As protection against phages, bacteria can modify cell surface structures that greatly enhance cell wall plasticity or cell envelope malleability, which impedes viral binding [63]. Dynamic cell surface fluxes can also provide “masking”, so metazoan immune cells cannot recognize and attach to bacterial pathogens. Masking can also provide protection against grazing protists. Therefore, immune phagocytes are less able to remove invading bacteria nor alert the other arms of the immune system, which prevents a coordinated defense by a host against an ongoing infection [63]. Some *Vibrio cholerae* isolates can use phase variation to alter the O-specific polysaccharide, a major target for host immune systems, in the outer membrane for phage evasion [68]. *Vibrio cholerae* can use an O-specific polysaccharide that continually varies to sustain host infection and transmission. Further information on how viruses can benefit the Vibrionaceae can be found in a recent review [16].

Grazing protists positively select for increased virulence in bacteria, because bacterivorous protists and immune cells from multicellular hosts kill bacteria in similar ways. In fact, there is accumulating evidence and growing scientific recognition that some bactericidal mechanisms involved in grazing protists and immune phagocytes are evolutionarily conserved [56,62]. However, investigating this topic is a complicated and convoluted undertaking, as the bactericidal machineries of predatory protists and multicellular host immunity also contain features that are unique and independent to each. In addition, not all the molecular, biochemical, physiological, and evolutionary details are fully understood. Surprising revelations are continually being found. Remarkably, recognition receptors for microbe-associated molecular patterns and interferon-γ-inducible responses in mammalian macrophages have homologs in amoebas [69]. The complexity on the topic (bactericidal processes in grazing protists and multicellular host immunity) is further magnified by the fact that grazing predators and host immune systems are not evolutionary static entities themselves, as they both acquire counteradaptations in an attempt to overcome the cunning of elusive bacteria. A few ploys bacteria use to thwart grazing protists have already been mentioned. After being ingested by protists, some bacteria block fusion of lysosomes and peroxisomes with the phagosome in which they reside, so a phagolysosome never develops [61,62]. Other bacteria will escape from the phagosome into the protist cytosol. As a phagolysosome forms, a harsh pH (usually highly acidic) in this compartment will ensue to aid digestion for the eukaryotic cells [60]. Some bacteria present in a developing phagolysosome are able to negate the extreme pH that forms, while others are able to prevent a shift from neutral pH in the first place [69]. All these processes enable the bacteria to avoid digestion by protists. These schemes also increase the likelihood that the same bacteria will withstand digestion by immune phagocytes. A complete molecular and biochemical description of the innumerable ways bacteria can circumvent bacterivorous protists is beyond the scope of the current review. Still, one mechanism (heavy metal intoxication) is described in some detail later in this exposition (see section on intracellular niches and heavy metal toxicity). Clearly, research shows the Vibrionaceae is proficient at obstructing predatory protists, a result that was not always appreciated [60,70,71]. Thus far, our discussion on protist-bacteria interactions has focused on virulence factors and pathogen–host relationships. Nevertheless, a stimulating question is how much can protist-bacteria associations inform researchers about the mutualisms that occur between bacteria and multicellular hosts. Protists are known to form intimate symbioses with bacteria, where both partners benefit [69]. On this subject, the squid–*Vibrio* mutualism could prove useful for discovering “symbiosis” factors (see Microbial Experimental Evolution below).

Whether or not all bacterivorous predators can ultimately facilitate the evolution of virulence factors in animal pathogens is equivocal. *Bdellovibrio*, *Micavibrio*, *Daptobacter*, *Vampirococcus*, and myxobacteria are all examples of voracious microbial predators [72], collectively known as *Bdellovibrio*-and-like organisms (BALO) microbes [73]. There is great interest in using these predatory microorganisms as biocontrol agents of pathogenic bacteria in agriculture, aquaculture, veterinary, and human medicine [74,75]. Metazoan immunity lacks the functional similarity with the modes of predation employed by BALO microorganisms that exists with protists (vacuolar/phagosomic bacterivory) [69]. These other bacterivorous predators are cytoplasmic, epibiotic, periplasmic, or social hunters [72]. Accordingly, BALO microbes might not evolutionarily enhance virulence in the same way phagosomic bacterivory does. However, some research suggests this is not a foregone conclusion. As with phagosomic bacterivory, BALO predation can select for increased biofilm/S-layer formation, elevated mucoidiness, and the rise of mutator genotypes [76,77,78], which can increase virulence. BALO hunters can also induce bacterial pathogens to evolve changes in enzyme activities, cell surface structures (masking), secretion systems, and the quorum sensing machinery as safeguards against predation that in themselves may eventually become virulence factors due to coincidental evolution [61,79,80]. For example, *Bdellovibrio* can prey on the Vibrionaceae, including *Vibrio* and *Photobacterium* [81,82]. For protection against *Bdellovibrio*, *Vibrio cholerae* can evolve modifications to its lipopolysaccharide and increase its motility; both of these traits are virulence factors in cholera [83]. Although protist grazing has been studied with *Vibrio fischeri* [70], how BALO microbes affect *Vibiro fischeri*’s ecology and evolution in the ocean or the squid–*Vibrio* mutualism has not been examined rigorously. Surely, the squid–*Vibrio* symbiosis is an exemplary study system to explore the manner BALO microorganisms shape host-microbe interactions, as a myriad of engaging research questions could be addressed. For instance, loss of the acetate switch protects *Vibrio vulnificus* from microbial predation [84], and the acetate switch is important for *Vibrio fischeri* colonization of the squid [85]. Thus, does a loss of function mutation for the acetate switch in *Vibrio fischeri* confer resistance to BALO predation? Perhaps more rivetingly, if *Vibrio fischeri* evolves resistance to BALO bacterivores, is there an accompanying loss of symbiosis competence with the squid?

## 4. Intracellular Niches & Resistance to Heavy Metal Toxicity

Certain taxonomic families are renown for their proficiencies to cycle between a free-living phase and an intracellular one within a eukaryotic host, such as the Legionellaceae, Mycobacteriaceae, and Rhizobiaceae [86,87,88]. Traditionally, the Vibrionaceae have not been seen in the like. However, many members of this bacterial family can adopt an intracellular lifestyle. The fish pathogen *Photobacterium damselae* is able to live within the macrophages of bass and also in epithelial cells of gilt-head bream [89,90]. *Vibrio anguillarum*, another fish pathogen, can also survive in salmon phagocytes [91]. *Vibrio ordalii* can live inside fish cells too [92]. *Vibrio shiloi* and *Vibrio coralliilyticus* can grow inside the cells of coral tissue [93,94]. *Vibrio cholerae* is able to inhabit and reproduce inside free-living amoebae, which could be one reservoir for this notorious pathogen [8,95]. *Vibrio parahaemolyticus* can propagate itself within human epithelial cells [96]. *Vibrio tasmaniensis* can reside within oyster hemocytes [63]. *Vibrio vulnificus* strains having an outer polysaccharide capsule might also be able to persist in oyster hemocytes [97]. *Vibrio harveyi* is an endosymbiont of *Cryptocaryon irritans*, a ciliated protist [98]. Resultantly, many case studies exist of the Vibrionaceae being adept at occupying intracellular niches. Strikingly, many previously unidentified and uncultured *Vibrio* spp. await discovery that are intracellular inhabitants of amoebae [99]. *Vibrio fischeri* cells have been observed in squid hemocytes, but an intracellular life stage is not thought to be an intrinsic part of the squid–*Vibrio* mutualism [100]. Rather, *Vibrio fischeri*’s strategy seems to be preferentially released (relative to nonsymbiotic bacteria like *Vibrio harveyi*) by the squid hemocytes, once the bacteria have been bound by the immune cells. That is, *Vibrio fischeri* aims to avoid engulfment by the squid hemocytes instead of trying to resist digestion or degradation [101]. Essentially, the squid hemocytes “choose” to release *Vibrio fischeri* cells after the bacteria have been “captured” by the immune cells in preparation for phagocytic digestion. The *Vibrio fischeri* outer membrane protein OmpU may partially mediate this process [102].

One mode for bacteria to repel intracellular killing by eukaryotic cells is to withstand or curb heavy metal toxicity, often copper and zinc [86]. Eukaryotic cells will frequently overload the phagosome (food vacuole) with these two metals after engulfing bacteria. Some intracellular bacteria will actively disrupt the processes by which eukaryotic cells uptake, accumulate, and concentrate heavy metals into their various compartments (e.g., organelles, cytosol, and endomembrane system), including ones involved in digestion like lysosomes and peroxisomes [63]. Moreover, intracellular bacteria can also produce chelating substances, which bind metal cations to remove them from the soluble milieu [63]. Bacteria can also erect exopolymeric structures—glycocalyx, slime layer, capsule, exopolysaccharide, etc.—that act as barriers to metal entry or serve as protective armor [103]. With the Vibrionaceae, modifications to the lipopolysaccharide layer or the cell envelope can function in this manner [63]. Volatilization and bioaccumulation (production of inclusions) are heavy metal detoxification mechanisms present in environmental bacteria [103,104], but their roles in intracellular survival of eukaryotic cells are ambiguous. These detoxification mechanisms have been described in *Pseudomonas*, which is capable of occupying an intracellular niche [105,106]. Another scheme is to convert the heavy metal to a less toxic state (i.e., biotransformation); conducting redox reactions to change the oxidation state is one avenue [103]. Another tactic is to precipitate the heavy metal, so it is not available for the intent of the eukaryotic cell. *Mycobacteria* and *Klebsiella* have both demonstrated resistance to heavy metals via insolubilization [107,108]. Both of these genera include intracellular parasites [86,109,110]. Case in point, ZnS and CuS are both highly insoluble salts. Bacteria can utilize sulfur metabolism, which might include H_2_S gas production or biochemical usage of the amino acids cysteine and methionine, to remove zinc and copper cations from solution. Since eukaryotic cells attempting to digest intracellular bacteria will readily concentrate phagosomes with copper, the internalized bacteria will sometimes express heavy metal efflux pumps and active transporters as countermeasures. For example, upon being engulfed by oyster hemocytes, *Vibrio tasmaniensis* will upregulate the expression of copper efflux genes [111].

Metals other than copper and zinc can be used by eukaryotic cells for the metal intoxication of internalized bacteria [112,113]; manganese and magnesium are other possibilities [114,115]. Evidence suggests some slime molds may use Mg^2+^ during grazing to kill certain prokaryotes serving as prey. [Some authorities consider magnesium a “light” metal [116].] Curiously, metals normally considered “benign” such as iron and potassium can also be used by eukaryotic cells to intoxicate or incapacitate intracellular prokaryotes under special circumstances [115]. Through Fenton reactions, “innocuous” metal cations like Fe^2+^ and K^+^ can be used by eukaryotes to create toxic reactive oxygen and nitrogen species that are harmful to intracellular bacteria. Such reactive species (superoxide anion, nitric oxide, free radicals, etc.) can form when there is a sudden influx or efflux of a benign metal cation into or from a eukaryotic intracellular compartment, like a vacuole or phagosome. When there is an accompanying abrupt change in pH, electrical charge, or temperature (exergonic reactions) in such a compartment, a devasting blow can be delivered to any residing bacteria [115]. K^+^ might at least be partly associated with inflammasome activation in macrophages or neutrophils this way. Bacteria can express loci (e.g., catalase and denitrifying enzymes) that blunt the antimicrobial effects of toxic reactive oxygen and nitrogen species, which can be created by the metallophysiology of a eukaryotic cell [115,117]. Some researchers hypothesize that immune phagocytes might purposely alternate between “benign” and “toxic” metal processes in the development of a phagosome as an enhanced design to stun and overwhelm engulfed bacteria. Apparently, certain intracellular parasites (*Mycobacterium*) have evolved sophisticated adaptations to counteract these cyclical “benign” and “toxic” metal actions that may sequentially occur in a phagosome [115]. Future research should determine whether the Vibrionaceae also possess such intricate adaptations. Additional metal toxicity countermeasures that have been identified in other taxonomic bacterial families include changes in cell morphology (surface-to-volume changes) and blebbing [103]. *Vibrio tasmaniensis* is capable of secreting outer membrane vesicles, when inside the phagosomes of oyster hemocytes, which might also provide resistance to metallotoxicity [63].

## 5. Agarases

The utilization of agar as a carbon source is uncommon in the microbial world, which is why it is the most widely used gellant or thickener for the preparation of solid culture media, Petri plates or slant tubes for example [118]. Agar (a mixture of agarose and agaropectins) is extracted from red algae (rhodophytes) for commercial use at an industrial scale, including culinary/food applications, cosmetics, pharmaceuticals, and scientific research (e.g., microbiology, molecular biology, and biotechnology). Moreover, novel and ever-growing usages are quickly emerging in medical, biomaterials, agriculture, biodegradable plastics, papermaking, and numerous other fields [119]. There has also been a burgeoning interest in the fermentation of agar for the production of economically useful products, including biofuels [120,121] and alcoholic beverages—using red algae for the production of “alternative” beers [39]. By substituting or supplementing agar polysaccharide (as an adjunct) for typically malted grains in brewing, the flavors, aromas, and textures (mouthfeel) of alcoholic beverages can be diversified and marketed to consumers, which can be a major goal of craft breweries [122]. For most red algae harvested, the fraction of agarose in agar is typically about 70%, but this value may fluctuate substantially 50–90%, [123]. For years, numerous papers were published documenting instances of agarase activity in the Vibrionaceae, but few of these reports ever identified isolates to the species level. Additionally, molecular characterization (e.g., 16S rRNA or DNA-DNA hybridization) was not always implemented to confirm the taxonomic identity of the isolates. Consequently, these early reports of positive agarase activity in the Vibrionaceae were suspect, as incertitude lingered over the taxonomic status of the microorganisms being investigated [118,124]. Thus, the true prevalence of agarolytic metabolism in the Vibrionaceae was rather unclear for a time. However, well characterized agar-hydrolyzing isolates have been uncovered recently, including *Vibrio agarivorans*, *Vibrio astriarenae*, *Vibrio algivorus*, *Vibrio sagamiensis*, and *Photobacterium swingsii* [125,126,127].

Interestingly, agarases play an important role in host-microbe interactions [128]. For red algae that possess agar as a constituent of their cell walls, agarases can trigger a host immune response. Agarolytic microorganisms will begin producing agarases, when they colonize or infect red algae. The agarases will then start to hydrolyze agar into various agaro-oligosaccharides and simpler sugars, such as neoagarobiose, 3, 6-anhydro-L-galactose, and D-galactose [129,130]. These simpler sugars, which are the building blocks and monomers of agar, elicit tenacious immune defenses and activate wound healing cascades within the red algal host [128]. Hence, agaro-oligosaccharides alert rhodophyte physiology that a microbial infection and/or injury might be imminent. Since processes in macroalgal immunity and would healing are poorly understood, many working hypotheses or conceptual models are borrowed from plant biology as a starting point [131,132]. Furthermore, red algal immunity is especially obscure. Unlike the case for phaeophytes and chlorophytes, the situation has been historically exacerbated with the lack of a good model system for the study of red algae, particularly for agarophytes [133,134,135]. The recent cultivation of *Gracilaria* with new emerging tools in molecular biology and bioinformatics has begun to illuminate agar metabolism and immunology within macroalgal rhodophytes [119,129].

As multicellular organisms, red algae possess a complex repertoire of physiological responses to address parasite threats or the trauma that pathogens can cause [136,137]. For instance, microbial agarases induce a respiratory oxidative burst in red algae that releases toxic reactive oxygen species that include antimicrobial peroxides, peroxols, and superoxides [128]. Additionally, halide peroxidases are upregulated that produce hypothiocyanous acid, hypohalous acids, and halogen free radicals, which also inhibit microbial growth [131,138,139]. Products of halide peroxidases are also known to disrupt quorum sensing and biofilm formation in microorganisms [132]. In some red algae, microbial degradation of agar can also promote the production of reactive nitrogen species (e.g., nitric oxide and peroxynitrous acid), which can combat pathogens by subjecting them to nitrosative stress [131,140,141]. Nitric oxide also serves as a signaling molecule in algal physiology, including in immunity, wound healing, and stress response [142,143]. Agar catabolism by microorganisms can also stimulate the endomembrane system and vesicular transport pathways in macrooalgae, with an upsurge in endocytosis and exocytosis [128]. Thus, the endoplasmic reticulum and Golgi apparatus become more bustling [144]. Moreover, the production and activity of lysosomes, peroxisomes, phagosomes, and vacuoles greatly increase.

In metazoan immunity, respiratory bursts unleashing oxidative and nitrosative stress upon parasites are typically done by specialized hemocytes or white blood cells [145]. These are immune cells that patrol an entire animal’s body against pathogens; they are also involved in mending injuries and carrying out tissue repair. The remodeling of the endomembrane system and vesicular trafficking is also reminiscent of these same functionally dedicated cell types, including macrophages, mastocytes, eosinophils, and neutrophils. Some of these patrolling immune cells, which prowl and roam throughout an individual’s body, fight microbial infections by gobbling up pathogens through endocytosis and phagocytosis [145]. As aforementioned, plant immunology serves as surrogate or proxy for processes that could be operating in algae, since much more data from long established model systems are available. Plants do not possess such roaming and patrolling immune cells due to their rigid cell wall structure, which is made of cellulose, nor do they possess specialized immune phagocytes [146,147]. However, plant cells are able to engulf pathogens, toxins, and parasite effector molecules via a modified mechanism involving autophagy—sometimes termed “xenophagy” or “heterophagy” [148,149]. Since the ability for plants to digest microorganisms has been most frequently documented in roots, it has been alternatively called “rhizophagy” [150,151]. The presence of specialized peripatetic phagocytes in macroalgae is uncertain; however, macroalgae are definitely capable of xenophagic engulfment of pathogens [152]. Growing evidence suggests that microbial catabolic products of agar can spark signaling transduction cascades in red algae, where the endomembrane system is galvanized to ingest parasites by xenophagic engulfment, perhaps into a digestive vacuole [128,144]. Additionally, algal vesicles carrying antimicrobial substances and digestive enzymes are directed to localized sites where an active infection is underway, as the host attempts to mount an immune response to fight back against unwelcomed microorganisms. Healing factors (e.g., callose synthesis) are also summoned to these areas [131,144]. Nonetheless, agar metabolism appears to be an evolutionary arms race between red algal hosts and agarase-producing microorganisms. Remarkably, some catabolic products of agar that are produced by microbial agarases might actually suppress certain aspects of red algal immunity. Notably, agarases are capable of generating a large diversity of hydrolytic products, especially when operating in conjunction with other classes of enzymes like glyco-stereoisomerases [118,130]. Recent research in laboratory animals and metazoan cell/tissue cultures shows that some oligosaccharides and simple sugars derived from agar catabolism can have bioactive properties, including antioxidant, anti-inflammatory, and anti-cancer (anti-mitotic) [153,154]. Hence, there is great interest in agar hydrolytic products for pharmaceutical, prebiotic, and nutraceutical applications.

Nevertheless, microbial agarases can generate products that neutralize the oxidative and nitrosative respiratory bursts produced by red agal hosts [131]. Likewise, agaro-oligosaccharides and agar-derived simple sugars can potentially scavenge halogen free radicals produced by halide peroxidases, which would nullify another branch of red algal immunity against pathogens [118,153,154]. “Anti-cancer” agaro-oligosaccharides can be mitosis inhibitors or interfere with the cell-division cycle in red algae, which can hinder tissue repair and regeneration. Hence, injuries, lesions, and trauma are not mended, and these wounds remain susceptible to further aggravation and infection. For example, callose deposition is associated with cytokinesis [155]. Agaro-oligosaccharides acting as cytokinesis inhibitors could prevent callose deposition from successfully ameliorating cell or tissue damage [156,157]. Similarly, agar-derived sugars that are toxins of cytoskeleton function would impede redeployment of the algal endomembrane system against invading pathogens. While an inflammatory response per se with the cardinal signs of redness, swelling, heat, and pain is absent from plants (and algae [158]), plant immunity does possess many components and processes that are akin to metazoan inflammation. Plants have a “resistosome”, where animals have an “inflammasome” [159]. Furthermore, plants utilize a “hypersensitive reaction”, where animal immunity implements “pyroptosis” [160]. Within metazoans, signaling molecules, cytokines, and chemokines direct inflammation in response to the detection of microbial-associated molecular patterns (e.g., lipopolysaccahride), damage-associated molecular patterns (e.g., extracellular ATP from ruptured host cells or molecular debris from a dismantled extracellular matrix), or danger signals, such as hydrophobic molecules that have been oxidized and catabolized (“hyppos”) [161]. Plant immunity can respond to these same alarmins.

Accordingly, many aspects of plant immunity and metazoan inflammation have functional equivalents. In some cases, the same signaling molecules or second messengers are even used in plants and animals for similar or analogous purposes, demonstrating an ancient aspect of immunity that is highly conserved [160,161]. Presumably, algal immunity (including agarophytes) also harbors some overlap with the animal inflammatory response: eicosanoids, prostaglandins, apoptotic caspases, and salicylic acid just to name a few [138,160,162]. Therefore, agar catabolic products that were “anti-inflammatory” in animals via mechanisms involving any of these constituents might also stifle red algal immunity and healing against microbial infections. Evolutionarily, red algae can attempt to counter pernicious microbial agarases by modifying the agar polymer in the cell wall with alternative sugar moieties (e.g., glucuronic acid and D-xylose) and substitutive functional groups—pyruvate, sulfate, and methoxy in place of alcohol—which creates a substrate that is a moving target for agarolytic enzymes [163]. To this end, the remodeling of agaropectins is a superb strategy, given its higher innate heterogeneity relative to agarose [164]. The exact biochemical inner workings that function here are obscure, as much still remains a mystery about agar biosynthesis in red algae [165]. Examining how agar catabolism shapes host-microbe associations between the Vibrionaceae and red algae is a fascinating endeavor worth pursuing. Such effects are likely to cascade to higher trophic levels and impact community ecology at large [166,167].

## 6. Phototrophy

In recent years, evidence for phototrophy within the Vibrionaceae has been discovered, including in the genera *Vibrio* and *Photobacterium* [168,169]. For instance, *Vibrio campbellii* BAA-1116 possesses proteorhodopsin [169], but not all isolates of this species are prototrophic, since some (*Vibrio campbellii* CAIM 333) lack the locus encoding this protein [170]. Phototrophic Vibrionaceae have been examples of photoheterotrophy or mixotrophy [171]. Hence, phototrophic Vibrionaceae still require organic compounds to build new biomass as they are not autotrophic. The identification of phototrophic Vibrionaceae has been an intriguing result, as this family has not been known for its ability to subsist off sunlight. The Vibrionaceae are typically viewed as chemoorganotrophs. Within the Vibrionaceae, phototrophy has thus far been associated with proteorhodopsin and appears to be the result of horizontal gene transfer. Additionally, the phototrophy has also been linked with enhanced ability to tolerate environmental stress, including carbon starvation and iron limitation [168,172]. Another important recent revelation has been that proteorhodopsin phototrophy can increase anaplerosis, which are biochemical reactions that “restock” a key metabolic pathway, the citric acid cycle for example [173]. Due to homeostasis, when microorganisms experience physiological stress, intermediate metabolites from central metabolic pathways will often be diverted or reallocated to specific cellular processes. For instance, particular catabolic and anabolic reactions will be initiated, stimulated, or shifted. Moreover, central intermediate metabolites might also be consumed due to stress responses being activated, including the stringent response as a riposte to nutrient starvation or resource limitation [173].

Hence, anaplerotic reactions refill or replenish central metabolic pathways with the integral intermediates necessary to keep them functional. In marine bacteria, the stimulation of proteorhodopsin phototrophy is coupled to the regulation of central metabolic pathways. As a result, proteorhodopsin phototrophy allows marine microbes to better scavenge trace levels of dissolved organic carbon and other nutrients in oligotrophic environments [173]. Not only does this ability promote survival in exiguous conditions, but it also maximizes the capacity for bacteria to successfully confront other concomitant environmental challenges (e.g., extreme temperature fluctuations) that might simultaneously be present when nutrients (iron, nitrogen, phosphorus, etc.) are especially limiting. Hence, anaplerosis could be a defining role of proteorhodopsin phototrophy. Consequently, photoheterotrophic Vibrionaceae would have increased flexibility in carbon acquisition pathways to efficiently adjust their biosynthetic machinery to natural fluctuations in light, limiting nutrients, and other environmental factors like pH and salinity [173]. Take for instance oligotrophic environments, if the citric acid cycle and the glyoxylate shunt are relieved from their duties for energy generation and the production of “reducing power” via catabolism, since light fulfills these tasks, the aforementioned biochemical pathways are more available for biosynthesis and cell growth for what little dissolved organic carbon is available [174,175]. Many proteorhodopsin phototrophic microbes engage in host-microbe interactions. For example, they are common residents of soft corals [176]. Furthermore, anaplerosis is known to influence host-microbe interactions [177,178]. Less is known about the interplay between these two phenomena in shaping host-microbe associations. As a result, studying what synergistic forces might emerge between proteorhodopsin phototrophy and anaplerosis to impact host-microbe relationships is a fascinating research question that merits further investigation [178]. As of yet, no *Vibrio fischeri* strains have been identified with proteorhodopsin.

## 7. Phage Shock Protein Response

The phage shock protein (PSP) response was first identified in *Escherichia coli* [179]. The loci governing the PSP response are organized as an operon (*pspABCDEFG*). The exact number of loci present in the operon depends on the bacterial species, and some taxa lack an intact PSP operon [180,181]. How the loci are organized (e.g., gene order) and regulated during gene expression can also vary among different species. Nonetheless, a general feature is that *pspA* exhibits the highest level of expression during operon upregulation. The locus *pspA* encodes for a protein which binds at the cell surface where fissures appear during stress [182,183]. Hence, the protein PspA serves as a sealant or caulk for immediately mending breaches that appear at the cell exterior when the structural integrity is being strained. The PSP response is not just a physiological counter to phage infections, as it can be stimulated by other environmental stressors, such as heat and hyperosmostic shock [184]. However, the PSP response is still distinct from these two stress responses, including the specific molecular chaperones induced. Much has been learned about the PSP response, but this stress response remains an enigma [181]. Many of the early null mutants that were first characterized in the PSP operon only displayed subtle phenotypes. Additionally, the exact inducing stimulus still has not been identified with absolute certainty.

Perturbations in the cell envelope, disruption in proton motive force, and changes in the membrane potential (i.e., transmembrane voltage) were early hypotheses as sparks for the activation of the PSP operon. Membrane elastic stress and redox state of the quinone pool are other possibilities [181]. Errors with proteins at the bacterial cell surface are other strong candidates—mislocalizations, misfolding, incorrect tertiary/quaternary structures, etc. The precise function of the PSP response is also unclear. Maintaining cytoplasmic membrane or cell envelope integrity during stress is surely one function [181,184]. Thus, there is general agreement that preventing cell leakage is one central purpose. The PSP response has been implicated in host-microbe interactions, including virulence in *Yersinia enterocolitica* and *Salmonella enterica* [181]. The PSP operon is present within the Vibrionaceae, but its role in host-microbe interactions is unclear. In one study, the PSP response was initiated in *Photobacterium damselae* subjected to antimicrobial peptides, but no host-microbe relationships were examined in this work [185]. In *Vibrio cholerae*, the PSP response was associated with virulence in zebrafish but not in mice [186]. *Vibrio fischeri* is known to have a PSP operon, but the PSP response’s role in colonizing the sepiolid squid has not yet been examined. The squid–*Vibrio* mutualism is an excellent model system to investigate the role of the PSP response in host-microbe relationships. In this regard, analyzing if the PSP response has any bearing on bioluminescence or quorum sensing in *Vibrio fischeri* would be worthwhile.

## 8. Microbial Experimental Evolution

With microbial experimental evolution, an investigator begins with an ancestral population and is able to observe the adaptations that occur in the descendent lineages under various selection schemes [10]. Microbial selection studies can be conducted under controlled and reproducible conditions to examine evolution, usually in the laboratory and on model organisms. Contrary to classical evolutionary analyses, where comparative or historical (e.g., phylogenetics) approaches are pursued, no assumptions in environmental conditions, the selection pressures involved, or in the ancestral and evolving populations are necessary, since these are controlled by the researcher. Experimental evolution permits tractability for the study of evolutionary biology by allowing experiments to be manipulated and repeated with replication [10]. Thus, microorganisms can be serially passaged under a particular selection regime for hundreds or even thousands of generations. Bacteria, including Vibrionaceae, are exemplary for such investigations. For instance, microbes have short doubling times that grant evolution and adaptation to be discernible on a human timespan [10]. Microorganisms also usually reach large population sizes in the settings that selection studies are performed, providing substantial opportunity for rare beneficial mutations to appear and achieve fixation by natural selection. What is more, deleterious mutations are expected to be purged (go extinct), since genetic drift is negligible in sizable populations. With microbes, evolving lineages can be stored in a −80⁰C freezer at varied evolutionary time points to construct a “frozen fossil record” [15]. The derived populations can later be reawakened from deep stupor and then be directly competed against the original (“unevolved”) ancestor or other evolutionary time points to determine relative fitness within any environment, including the ancestral one or the selection regime (derived conditions). For example, a derived lineage that has sustained evolutionary adaptation in response to a specific selection pressure for 1000 generations could be competed against the 750-, 500-, and 250-generations time points, or even the original “0 generations” ancestor. These competitions could be completed in the ancestral environment to determine if evolutionary tradeoffs had accrued during adaptation to a novel environment [15]. The “frozen fossil record” also enables an investigator to ascertain the evolutionary episode that a novel adaptive trait first arose. Experimental evolution can also effectively model stochastic variation as well. For instance, genetic polymorphisms that arise can be analyzed to see if they are maintained by balancing selection or whether the diversity is mostly a neutral transient phase of molecular evolution (“neutralist–selectionist” debate) [187]. Even the evolution of mutation rates as polymorphisms can be analyzed [188,189].

Interestingly, microbial selection experiments may be “replayed” from various time points to see if ensuing evolutionary trajectories are contingent on prior genetic changes or previously modified traits [190]—historical contingency versus determinism (natural selection). Furthermore, ancestral and derived lineages can be surveyed afterward to resolve what mutations or genetic alterations have occurred and which are responsible for novel adaptive traits [7,191]. Microbial experimental evolution, too, allows one to perform thought-provoking and extraordinary experiments that would be impossible under normal circumstances: Imagine being able to compete cuttlefishes against ammonites, which became extinct at the end of the Cretaceous 66 million years ago when a colossal asteroid collided with Earth [192]. Two cephalopods from different geological ages, which would dominate as great hunters the benthic regions or pelagic zones of contemporary oceans? Which would prevail in oceans of the Cretaceous, or even the Ordovician, which ended almost 444 million years ago? Alternatively, envision having woolly mammoths clash against elephants. Which would rule the steppes and forests? These showdowns of fitness can also be done with primates. Who would win a chess game or an arm wrestling contest between *Homo erectus* and *Homo sapiens sapiens* (modern humans)? Who would throw a javelin better? Truly, with microbial experimental evolution, such analogous grandeur competitions of relative fitness can be designed. Main event matches between “Titans of Natural History” become possible. Due to the “frozen fossil record”, a feature of microbial selection studies is the capacity to invoke enchanting and romantic imagery. For further illustration, another example is the idea of “replaying life’s tape”, which was a premise or thought experiment surmised by Stephen Jay Gould [193]. Gould envisaged the evolution of life on Earth being allowed to be “replayed”, from the Hadean Eon to the present-day, in countless (perhaps even infinite) iterations, where each was an independent trial run of the planet’s natural history. Gould asked rhetorically whether or not under such circumstances would human sentience or intelligence evolve again, or any other specific outcome for that matter. In other words, how reproducible is the evolutionary fallout that emerges in Earth’s history of life? Hence, Gould highlighted the roles chance, contingency, and determinism (natural selection/adaptation) all play in shaping the history of life [193].

Do reptilian dinosaurs always arise to dominate the land, air, and sea (e.g., ichthyosaurs), only to eventually be extinguished by an extraterrestrial collision and replaced by mammals in all three realms? In fact, perhaps neither reptiles nor mammals emerge 98 % of the time. Rather, gargantuan arthropods are the major animal group that invades Earth’s realms, with vertebrates almost never happening in most reiterations. Sometimes subsequent evolutionary events are highly dependent or “contingent” on previous ones. Do most scenarios on Earth follow this pattern instead, when “replaying life’s tape” repeatedly? Chaos theory has been proposed as a way to mathematically model such sensitive systems and evolutionary dynamics, where computer simulations creating strange attractors with fractal structure can simulate the multiple iterations of life’s history on Earth [194,195]. In such mathematical models, the butterfly effect can characterize evolutionary trajectories, cascades, and outcomes that are very susceptible to initial or prior conditions [196]. In any event, with microbial experimental evolution, alluring and riveting experiments (like Gould’s) can actually be performed [190,197]. In addition, experimental evolution with microorganisms can provide real empirical data to complement mathematical models and computer simulations, like in the illustration with chaos theory. For many years, the Vibrionaceae were largely absent from microbial selection studies [10]. This was a shameful loss to the study of evolutionary biology and microbiology, given all the major advantages the Vibrionaceae had to offer: tremendous genetic and metabolic diversity, marine bioluminescence, a complex quorum sensing machinery, a mammoth propensity to engage in host-microbe relationships, proficient biofilm formation, etc. Notwithstanding, the use of the Vibrionaceae in microbial selection studies is on the rise. *Vibrio campbellii* was used to study social evolution, cooperation, and cheater control [198]. The squid–*Vibrio* mutualism has enjoyed splendid success with microbial experimental evolution, as it has enlightened how a symbiont’s adaptation to stress during the free-living phase can affect host-microbe affiliations [13,15,199]. Even intriguing topics like adaptive radiation, island evolution, and biogeography theory have been examined [7,14]. The squid–*Vibrio* symbiosis has also demonstrated how biofilm evolution can increase *Vibrio fischeri*’s resistance to oxidative stress [191]. Unquestionably, the Vibrionaceae and the squid–*Vibrio* symbiosis will continue to be valuable in addressing many fascinating subjects.

## 9. Conclusions

The Vibrionaceae possesses tremendous genetic and metabolic diversity. The taxonomic group is ubiquitous in aquatic environments throughout the world, freshwater, brackish, and marine. Due to the bacterial family’s broad ecological niche breadth, ease of culturability in most cases, sequenced genomes available, and its malleability to molecular tools, the Vibrionaceae is ideal for addressing many different scientific topics and contexts, including basic and applied research. The Vibrionaceae is an outstanding model system and opportunity to investigate several phenomena, including biofilm formation, bioluminescence, quorum sensing, and the entire spectrum of host-microbe relationships—symbioses, commensalisms, and parasitisms (pathogenicities). Nevertheless, there are still many areas where little is known about the Vibrionaceae. For instance, compared to what is known about its affiliation with other eukaryotic hosts, the information available on the Vibrionaceae’s ecological and evolutionary connection to marine plants is scant. Filling in this knowledge gap will be crucial for applications in agriculture, zymology, conservation biology, and managing climate change. For similar reasons, identifying and characterizing more Vibrionaceae isolates that produce agarases is important, as agarophytic red algae stand at the cusp of becoming a major crop (especially in Asia) for the food, pharmaceutical, nutraceutical, and fermentation industries, along with biotechnology. Vibrionaceae agarases will also be valuable for a better comprehension of immunology. Much has been learned about the role of microbial predation in facilitating virulence in bacteria, including the Vibrionaceae. Similarly, intracellular niches and resistance to heavy metal toxicity can raise the pathogenicity of bacteria. Additional inquiries are needed in these fields. The PSP response continues to somewhat baffle researchers, as the exact function is still under lively discussion. Many details have been deciphered, but this stress response pathway remains to a certain degree a mystery. Conceivably, the PSP response may even be involved in some way with many of the other sections discussed in this current review, host-microbe associations with seagrasses, intracellular niches, or resistance to heavy metals for example. The squid–*Vibrio* symbiosis is especially promising for studying the role of the PSP response in host-microbe affiliations. The Vibrionaceae largely remains an untapped resource for microbial experimental evolution. A bonanza of wealth and riches awaits researchers who decide to invest and develop this taxon for microbial selection studies. The squid–*Vibrio* mutualism has proven this claim true.

## Data Availability

Not applicable.

## References

[1] Soto W., Lostroh C.P., Nishiguchi M.K., Seckback J., Grube M. (2010). Physiological responses to stress in the *vibrionaceae*. Cooperation and Stress in Biology.

[2] Pulliam H.R. (1988). Sources, sinks, and population regulation. Am. Nat..

[3] Sawabe T., Sugimura I., Ohtsuka M., Nakano K., Tajima K., Ezura Y., Christen R. (1998). *Vibrio halioticoli* sp. nov., a non-motile alginolytic marine bacterium isolated from the gut of the abalone *Haliotis discus hannai*. Int. J. Syst. Evol. Microbiol..

[4] Shieh W.Y., Chen A.L., Chiu H.-H. (2000). *Vibrio aerogenes* sp. nov., a facultatively anaerobic marine bacterium that ferments glucose with gas production. Int. J. Syst. Evol. Microbiol..

[5] Hendry T.A., Freed L.L., Fader D., Fenolio D., Sutton T.T., Lopez J.V. (2018). Ongoing transposon-mediated genome reduction in the luminous bacterial symbionts of deep-sea ceratioid anglerfishes. MBio.

[6] Hendry T.A., De Wet J.R., Dougan K.E., Dunlap P.V. (2016). Genome evolution in the obligate but environmentally active luminous symbionts of flashlight fish. Genome Biol. Evol..

[7] Soto W., Travisano M., Tolleson A.R., Nishiguchi M.K. (2019). Symbiont evolution during the free-living phase can improve host colonization. Microbiology.

[8] Abd H., Weintraub A., Sandström G. (2005). Intracellular survival and replication of *Vibrio cholerae* O139 in aquatic free-living amoebae. Environ. Microbiol..

[9] Ringø E., Li X., Van Doan H., Ghosh K. (2022). Interesting probiotic bacteria other than the more widely used lactic acid bacteria and bacilli in finfish. Front. Mar. Sci..

[10] Soto W., Nishiguchi M.K. (2014). Microbial experimental evolution as a novel research approach in the vibrionaceae and squid-*Vibrio* symbiosis. Front. Microbiol..

[11] Burgess J.G., Thompson F.L., Austin B., Swings J. (2006). Biotechnological applications. Biology of Vibrios.

[12] Soto W., Gutierrez J., Remmenga M.D., Nishiguchi M.K. (2009). Salinity and temperature effects on physiological responses of *Vibrio fischeri* from diverse ecological niches. Microb. Ecol..

[13] Cohen M.L., Mashanova E.V., Jagannathan S.V., Soto W. (2020). Adaptation to pH stress by *Vibrio fischeri* can affect its symbiosis with the Hawaiian bobtail squid (*Euprymna scolopes*). Microbiology.

[14] Soto W., Rivera F.M., Nishiguchi M.K. (2014). Ecological diversification of *Vibrio fischeri* serially passaged for 500 generations in novel squid host *Euprymna tasmanica*. Microb. Ecol..

[15] Cohen M.L., Mashanova E.V., Rosen N.M., Soto W. (2019). Adaptation to temperature stress by *Vibrio fischeri* facilitates this microbe’s symbiosis with the Hawaiian bobtail squid (*Euprymna scolopes*). Evolution.

[16] Soto W., Nishiguchi M.K. (2021). Environmental stress selects for innovations that drive *Vibrio* symbiont diversity. Front. Ecol. Evol..

[17] Zhou X., Sang W., Liu S., Zhang Y., Ge H. (2010). Modeling and prediction for the acute toxicity of pesticide mixtures to the freshwater luminescent bacterium *Vibrio qinghaiensis* sp.-q67. J. Environ. Sci..

[18] El-Son M.A.M., Elbahnaswy S., Ibrahim I. (2020). Molecular and histopathological characterization of *Photobacterium damselae* in naturally and experimentally infected Nile tilapia (*Oreochromis niloticus*). J. Fish Dis..

[19] Budsberg K.J., Wimpee C.F., Braddock J.F. (2003). Isolation and identification of *Photobacterium phosphoreum* from an unexpected niche: Migrating salmon. Appl. Environ. Microbiol..

[20] Reusch T.B.H., Schubert P.R., Marten S.-M., Gill D., Karez R., Busch K., Hentschel U. (2021). Lower *Vibrio* spp. abundances in *Zostera marina* leaf canopies suggest a novel ecosystem function for temperate seagrass beds. Mar. Biol..

[21] Ettinger C.L., Eisen J.A. (2020). Fungi, bacteria and oomycota opportunistically isolated from the seagrass, *Zostera marina*. PLoS ONE.

[22] Bagwell C.E., La Rocque J.R., Smith G.W., Polson S.W., Friez M.J., Longshore J.W., Lovell C.R. (2002). Molecular diversity of diazotrophs in oligotrophic tropical seagrass bed communities. FEMS Microbiol. Ecol..

[23] Franco A., Rückert C., Blom J., Busche T., Reichert J., Schubert P., Goesmann A., Kalinowski J., Wilke T., Kämpfer P. (2020). High diversity of *Vibrio* spp. associated with different ecological niches in a marine aquaria system and description of *Vibrio aquimaris* sp. nov. Syst. Appl. Microbiol..

[24] Mansson M., Gram L., Larsen T.O. (2011). Production of bioactive secondary metabolites by marine vibrionaceae. Mar. Drugs.

[25] Frans I., Michiels C.W., Bossier P., Willems K.A., Lievens B., Rediers H. (2011). *Vibrio anguillarum* as a fish pathogen: Virulence factors, diagnosis and prevention. J. Fish Dis..

[26] Parmar P., Shukla A., Goswami D., Gaur S., Patel B., Saraf M. (2020). Comprehensive depiction of novel heavy metal tolerant and eps producing bioluminescent *Vibrio alginolyticus* pbr1 and *V. rotiferianus* pbl1 confined from marine organisms. Microbiol. Res..

[27] Sullivan B.K., Trevathan-Tackett S.M., Neuhauser S., Govers L.L. (2018). Review: Host-pathogen dynamics of seagrass diseases under future global change. Mar. Pollut. Bull..

[28] Ugarelli K., Chakrabarti S., Laas P., Stingl U. (2017). The seagrass holobiont and its microbiome. Microorganisms.

[29] Conte C., Rotini A., Manfra L., D’Andrea M.M., Winters G., Migliore L. (2021). The seagrass holobiont: What we know and what we still need to disclose for its possible use as an ecological indicator. Water.

[30] Tarquinio F., Hyndes G.A., Laverock B., Koenders A., Säwström C. (2019). The seagrass holobiont: Understanding seagrass-bacteria interactions and their role in seagrass ecosystem functioning. FEMS Microbiol. Lett..

[31] Conte C., Rotini A., Winters G., Vasquez M.I., Piazza G., Kletou D., Migliore L. (2021). Elective affinities or random choice within the seagrass holobiont? The case of the native *Posidonia oceanica* (L.) delile and the exotic *Halophila stipulacea* (forssk.) asch. from the same site (limassol, cyprus). Aquat. Bot..

[32] Orth R.J., Carruthers T.J.B., Dennison W.C., Duarte C.M., Fourqurean J.W., Heck K.L., Randall H.A., Kendrick G.A., Kenworthy W.J., Olyarnik S. (2006). A global crisis for seagrass ecosystems. BioScience.

[33] Nguyen H.M., Ralph P.J., Marín-Guirao L., Pernice M., Procaccini G. (2021). Seagrasses in an era of ocean warming: A review. Biol. Rev..

[34] Douglas A.E., Werren J.H. (2016). Holes in the hologenome: Why host-microbe symbioses are not holobionts. MBio.

[35] Kim D.H., Mahomoodally M.F., Sadeer N.B., Seok P.G., Zengin G., Palaniveloo K., Khalile A.A., Rauf A., Rengasamy K.R.R. (2021). Nutritional and bioactive potential of seagrasses: A review. S. Afr. J. Bot..

[36] Ratnawati N.N., Jompa J., Rappe R.A. (2019). Fruits of *Enhalus acoroides* as a source of nutrition for coastal communities. Earth Environ. Sci..

[37] Coria-Monter E., Durán-Campos E. (2020). The seagrass *Syringodium filiforme* as a possible alternative for human consumption. Int. J. Agric. Food Sci. Technol..

[38] Uchida M., Miyoshi T., Kaneniwa M., Ishihara K., Nakashimada Y., Urano N. (2014). Production of 16.5% *v*/*v* ethanol from seagrass seeds. J. Biosci. Bioeng..

[39] Uchida M., Kim S.-K. (2015). Fermentation of seaweeds and its applications. Seafood Science: Advances in Chemistry, Technology and Applications.

[40] Abdulla R., Ariffin Z. (2016). Quantitative assessment of seagrass as bioethanol feedstock. Trans. Sci. Technol..

[41] Rajkumar J., Dilipan E., Ramachandran M., Panneerselvam A., Thajuddin N. (2021). Bioethanol production from seagrass waste, through fermentation process using cellulase enzyme isolated from marine actinobacteria. Vegetos.

[42] Ścieszka S., Klewicka E. (2019). Algae in food: A general review. Crit. Rev. Food Sci. Nutr..

[43] Uchida M., Kurushima H., Ishihara K., Murata Y., Touhata K., Ishida N., Niwa K., Araki T. (2017). Characterization of fermented seaweed sauce prepared from nori (*Pyropia yezoensis*). J. Biosci. Bioeng..

[44] Figueroa V., Farfán M., Aguilera J.M. (2021). Seaweeds as novel foods and source of culinary flavors. Food Rev. Int..

[45] Torres M.D., Kraan S., Domínguez H. (2019). Seaweed biorefinery. Rev. Environ. Sci. Bio/Technol..

[46] Gao Z.-M., Xiao J., Wang X.-N., Ruan L.-W., Chen X.-L., Zhang Y.-Z. (2012). *Vibrio xiamenensis* sp. nov., a cellulase-producing bacterium isolated from mangrove soil. Int. J. Syst. Evol. Microbiol..

[47] Deep K., Poddar A., Das S.K. (2016). Cloning, overexpression, and characterization of halostable, solvent-tolerant novel β-endoglucanase from a marine bacterium *Photobacterium panuliri* LBS5T (DSM 27646T). Appl. Biochem. Biotechnol..

[48] Iyapparaj P., Revathi P., Ramasubburayan R., Prakash S., Palavesam A., Immanuel G., Anantharaman P., Sautreau A., Hellio C. (2014). Antifouling and toxic properties of the bioactive metabolites from the seagrasses *Syringodium isoetifolium* and *Cymodocea serrulata*. Ecotoxicol. Environ. Saf..

[49] Yuvaraj N., Kanmani P., Satishkumar R., Paari A., Pattukumar V., Arul V. (2012). Seagrass as a potential source of natural antioxidant and anti-inflammatory agents. Pharm. Biol..

[50] Orhan I., Sener B., Atıcı T., Brun R., Perozzo R., Tasdemir D. (2006). Turkish freshwater and marine macrophyte extracts show in vitro antiprotozoal activity and inhibit FabI, a key enzyme of *Plasmodium falciparum* fatty acid biosynthesis. Phytomedicine.

[51] Regina C.M.P., Ahmadi P., Hertiani T., Septiana E., Putra M.Y., Chianese G. (2022). A comprehensive update on the bioactive compounds from seagrasses. Marine Drugs.

[52] Tarquinio F., Attlan O., Vanderklift M.A., Berry O., Bissett A. (2021). Distinct endophytic bacterial communities inhabiting seagrass seeds. Front. Microbiol..

[53] Petersen L.-E., Marner M., Labes A., Tasdemir D. (2019). Rapid metabolome and bioactivity profiling of fungi associated with the leaf and rhizosphere of the Baltic seagrass *Zostera marina*. Mar. Drugs.

[54] Blanchet E., Prado S., Stien D., Da Silva J.O., Ferandin Y., Batailler N., Intertaglia L., Escargueil A., Lami R. (2017). Quorum sensing and quorum quenching in the Mediterranean seagrass *Posidonia oceanica* microbiota. Front. Mar. Sci..

[55] Zieman J.C. (1982). The Ecology of the Seagrasses of South Florida: A Community Profile.

[56] Brüssow H. (2007). Bacteria between protists and phages: From antipredation strategies to the evolution of pathogenicity. Mol. Microbiol..

[57] Faruque S.M., Mekalanos J.J. (2012). Phage-bacterial interactions in the evolution of toxigenic *Vibrio cholerae*. Virulence.

[58] Davis B.M., Waldor M.K. (2003). Filamentous phages linked to virulence of *Vibrio cholerae*. Curr. Opin. Microbiol..

[59] Van Valen L. (1973). A new evolutionary law. Evol. Theory.

[60] Espinoza-Vergara G., Hoque M.M., McDougald D., Noorian P. (2020). The impact of protozoan predation on the pathogenicity of *Vibrio cholerae*. Front. Microbiol..

[61] Erken M., Lutz C., McDougald D. (2013). The rise of pathogens: Predation as a factor driving the evolution of human pathogens in the environment. Microb. Ecol..

[62] Sun S., Noorian P., McDougald D. (2018). Dual role of mechanisms involved in resistance to predation by protozoa and virulence to humans. Front. Microbiol..

[63] Destoumieux-Garzón D., Canesi L., Oyanedel D., Travers M.-A., Charrière G.M., Pruzzo C., Vezzulli L. (2020). *Vibrio*–bivalve interactions in health and disease. Environ. Microbiol..

[64] Karaolis D.K.R., Somara S., Maneval D.R., Johnson J.A., Kaper J.B. (1999). A bacteriophage encoding a pathogenicity island, a type-IV pilus and a phage receptor in cholera bacteria. Nature.

[65] Rowe-Magnus D.A., Zouine M., Didier Mazel D., Thompson F.L., Brian Austin B., Swings J. (2006). The adaptive genetic arsenal of pathogenic *Vibrio* species: The role of integrons. The Biology of Vibrios.

[66] Stoddard B.L. (2006). Homing endonuclease structure and function. Q. Rev. Biophys..

[67] Kidwell M.G., Lisch D.R. (2001). Perspective: Transposable elements, parasitic DNA, and genome evolution. Evolution.

[68] Seed K.D., Faruque S.M., Mekalanos J.J., Calderwood S.B., Qadri F., Camilli A. (2012). Phase variable o antigen biosynthetic genes control expression of the major protective antigen and bacteriophage receptor in *Vibrio cholerae* O1. PLoS Pathog..

[69] Amaro F., Martín-González A. (2021). Microbial warfare in the wild—the impact of protists on the evolution and virulence of bacterial pathogens. Int. Microbiol..

[70] Chavez-Dozal A., Gorman C., Erken M., Steinberg P.D., McDougald D., Nishiguch M.K. (2013). Predation response of *Vibrio fischeri* biofilms to bacterivorus protists/phagotrophic protozoa. Appl. Environ. Microbiol..

[71] Robino E., Poirier A.C., Amraoui H., Le Bissonnais S., Perret A., Lopez-Joven C., Auguet J.-C., Rubio T.P., Cazevieille C., Rolland J.-L. (2020). Resistance of the oyster pathogen *Vibrio tasmaniensis* LGP32 against grazing by *Vannella* sp. marine amoeba involves vsm and copa virulence factors. Environ. Microbiol..

[72] Madigan M.T., Bender K.S., Buckley D.H., Sattley W.M., Stahl D.A. (2021). Ecological Diversity of Bacteria. Brock Biology of Microorganisms 16th Edition.

[73] Williams H.N., Piñeiro S., Jurkevitch E. (2006). Ecology of the predatory *Bdellovibrio* and like organisms. Predatory Prokaryotes—Biology, Ecology and Evolution.

[74] Najnine F., Cao Q., Zhao Y., Cai J., Jurkevitch E., Mitchell R.J. (2020). Antibacterial activities of *Bdellovibrio* and like organisms in aquaculture. The Ecology of Predation at the Microscale.

[75] McNeely D., Chanyi R.M., Dooley J.S., Moore J.E., Koval S.F. (2016). Biocontrol of *Burkholderia cepacia* complex bacteria and bacterial phytopathogens by *Bdellovibrio bacteriovorus*. Can. J. Microbiol..

[76] Nair R.R., Vasse M., Wielgoss S., Sun L., Yu Y.-T.N., Velicer G.J. (2019). Bacterial predator-prey coevolution accelerates genome evolution and selects on virulence-associated prey defences. Nat. Commun..

[77] Koval S.F., Hynes S.H. (1991). Effect of paracrystalline protein surface layers on predation by *Bdellovibrio bacteriovorus*. J. Bacteriol..

[78] Kadouri D., O’Toole G.A. (2005). Susceptibility of biofilms to *Bdellovibrio bacteriovorus* attack. Appl. Environ. Microbiol..

[79] Dwidar M., Jang H., Sangwan N., Mun W., Im H., Yoon S., Choi S., Nam D., Mitchell R.J. (2021). Diffusible signaling factor, a quorum-sensing molecule, interferes with and is toxic towards *Bdellovibrio bacteriovorus* 109J. Microb. Ecol..

[80] Aharon E., Mookherjee A., Pérez-Montaño F., Da Silva G.M., Sathyamoorthy R., Burdman S., Jurkevitch E. (2021). Secretion systems play a critical role in resistance to predation by *Bdellovibrio bacteriovorus*. Res. Microbiol..

[81] Varon M. (1981). Interaction of *Bdellovibrio* with its prey in mixed microbial populations. Microb. Ecol..

[82] Cao H., Wang H., Yu J., An J., Chen J. (2019). Encapsulated *Bdellovibrio* powder as a potential bio-disinfectant against whiteleg shrimp-pathogenic vibrios. Microorganisms.

[83] Duncan M.C., Forbes J.C., Nguyen Y., Shull L.M., Gillette R.K., Lazinski D.W., Ali A., Shanks R.M.Q., Kadouri D.E., Camilli A. (2018). *Vibrio cholerae* motility exerts drag force to impede attack by the bacterial predator *Bdellovibrio bacteriovorus*. Nat. Commun..

[84] Regina V.R., Noorian P., Sim C.B.W., Constancias F., Kaliyamoorthy E., Booth S.C., Espinoza-Vergara G., Rice S.A., McDougald D. (2022). Loss of the acetate switch in *Vibrio vulnificus* enhances predation defense against *Tetrahymena pyriformis*. Appl. Environ. Microbiol..

[85] Studer S.V., Mandel M.J., Ruby E.G. (2008). AinS quorum sensing regulates the *Vibrio fischeri* acetate switch. J. Bacteriol..

[86] Neyrolles O., Wolschendorf F., Mitra A., Niederweis M. (2015). Mycobacteria, metals, and the macrophage. Immunol. Rev..

[87] De Castro C., Molinaro A., Lanzetta R., Silipo A., Parrilli M. (2008). Lipopolysaccharide structures from *Agrobacterium* and *Rhizobiaceae* species. Carbohydr. Res..

[88] Shin S., Roy C.R. (2008). Host cell processes that influence the intracellular survival of *Legionella pneumophila*. Cell. Microbiol..

[89] Elkamel A.A., Hawke J.P. (2003). *Photobacterium damselae* subsp. *piscicida* is capable of replicating in hybrid striped bass macrophages. J. Aquat. Anim. Health.

[90] Acosta F., Vivas J., Padilla D., Vega J., Bravo J., Grasso V., Real F. (2009). Invasion and survival of *Photobacterium damselae* subsp. *piscicida* in non-phagocytic cells of gilthead sea bream, *Sparus aurata* L.. J. Fish Dis..

[91] Larsen M.H., Boesen H.T. (2001). Role of flagellum and chemotactic motility of *Vibrio anguillarum* for phagocytosis by and intracellular survival in fish macrophages. FEMS Microbiol. Lett..

[92] Ruben Avendaño-Herrera R., Arias-Muñoz E., Rojas V., Toranzo A.E., Poblete-Morales M., Córdova C., Irgang R. (2019). Evidence for the facultative intracellular behaviour of the fish pathogen *Vibrio ordalii*. J. Fish Dis..

[93] Rosenberg E., Koren O., Thompson F.L., Austin B., Swings J. (2006). Vibrios in coral health and disease. Biology of Vibrios.

[94] Vidal-Dupiol J., Ladriere O., Destoumieux-Garzon D., Sautiere P.-E., Meistertzheim A.L., Tambutte E., Tambutte S., Duval D., Foure L., Adjeroud M. (2011). Innate immune responses of a scleractinian coral to vibriosis. J. Biol. Chem..

[95] Van der Henst C., Scrignari T., Maclachlan C., Blokesch M. (2016). An intracellular replication niche for *Vibrio cholerae* in the amoeba *Acanthamoeba castellanii*. ISME J..

[96] De Souza Santos M., Orth K. (2014). Intracellular *Vibrio parahaemolyticus* escapes the vacuole and establishes a replicative niche in the cytosol of epithelial cells. MBio.

[97] Harris-Young L., Tamplin M.L., Mason J.W., Aldrich H.C., Jackson J.K. (1995). Viability of *Vibrio vulnificus* in association with hemocytes of the American oyster (*Crassostrea virginica*). Appl. Environ. Microbiol..

[98] Qiao Y., Wang J., Mao Y., Liu M., Chen R., Su Y., Ke Q., Han K., Zheng W. (2017). Pathogenic bacterium *Vibrio harveyi*: An endosymbiont in the marine parasitic ciliate protozoan *Cryptocaryon irritans*. Acta Oceanol. Sin..

[99] MacPhail D.P.C., Koppenstein R., Maciver S.K., Paley R., Longshaw M., Henriquez F.L. (2021). *Vibrio* species are predominantly intracellular within cultures of *Neoparamoeba perurans*, causative agent of amoebic gill disease (agd). Aquaculture.

[100] Nyholm S.V., Stewart J.J., Ruby E.G., McFall-Ngai M.J. (2009). Recognition between symbiotic *Vibrio fischeri* and the haemocytes of *Euprymna scolopes*. Environ. Microbiol..

[101] McAnulty S.J., Nyholm S.V. (2017). The role of hemocytes in the Hawaiian bobtail squid, *Euprymna scolopes*: A model organism for studying beneficial host–microbe interactions. Front. Microbiol..

[102] Rader B., McAnulty S.J., Nyholm S.V. (2019). Persistent symbiont colonization leads to a maturation of hemocyte response in the *Euprymna scolopes*/*Vibrio fischeri* symbiosis. MicrobiologyOpen.

[103] Mathivanan K., Chandirika J.U., Vinothkanna A., Yin H., Liu X., Meng D. (2021). Bacterial adaptive strategies to cope with metal toxicity in the contaminated environment–a review. Ecotoxicol. Environ. Saf..

[104] Fakhar A., Gul B., Gurmani A.R., Shafaqat S.M.K., Ali T.S., Chaudhary H.J., Rafique M., Rizwan M. (2020). Heavy metal remediation and resistance mechanism of *Aeromonas*, *Bacillus*, and *Pseudomonas*: A review. Crit. Rev. Environ. Sci. Technol..

[105] Zhang J., Cao T., Tang Z., Shen Q., Rosen B.P., Zhao F.-J. (2015). Arsenic methylation and volatilization by arsenite s-adenosylmethionine methyltransferase in *Pseudomonas alcaligenes* NBRC14159. Appl. Environ. Microbiol..

[106] Penaranda C., Chumbler N.M., Hung D.T. (2021). Dual transcriptional analysis reveals adaptation of host and pathogen to intracellular survival of *Pseudomonas aeruginosa* associated with urinary tract infection. PLoS Pathog..

[107] Erardi F.X., Failla M.L., Falkinham J. (1987). Plasmid-encoded copper resistance and precipitation by *Mycobacterium scrofulaceum*. Appl. Environ. Microbiol..

[108] Aiking H., Govers H., Van’t Rie J. (1985). Detoxification of cadmium, mercury and lead in *Klebsiella aerogenes* NCTC418 growing in continuous culture. Appl. Environ. Microbiol..

[109] Cano V., March C., Insua J.L., Aguiló N., Llobet E., Moranta D., Regueiro V., Brennan G.P., Millán-Lou M.I., Martín C. (2015). *Klebsiella pneumoniae* survives within macrophages by avoiding delivery to lysosomes. Cell Microbiol..

[110] Bengoechea J.A., Pessoa J.S. (2019). *Klebsiella pneumoniae* infection biology: Living to counteract host defences. FEMS Microbiol. Rev..

[111] Vanhove A.S., Rubio T.P., Nguyen A.N., Lemire A., Roche D., Nicod J., Vergnes A., Poirier A.C., Disconzi E., Bachère E. (2016). Copper homeostasis at the host vibrio interface: Lessons from intracellular vibrio transcriptomics. Environ. Microbiol..

[112] Sheldon J.R., Skaar E.P. (2019). Metals as phagocyte antimicrobial effectors. Curr. Opin. Immunol..

[113] Botella H., Stadthagen G., Lugo-Villarino G., De Chastellier C., Neyrolles O. (2012). Metallobiology of host–pathogen interactions: An intoxicating new insight. Trends Microbiol..

[114] Hood M.I., Skaar E.P. (2012). Nutritional immunity: Transition metals at the pathogen–host interface. Nat. Rev. Microbiol..

[115] Soldati T., Neyrolles O. (2012). Mycobacteria and the intraphagosomal environment: Take it with a pinch of salt(s)!. Traffic.

[116] Brandes E.A., Brook G.B. (1998). General physical properties of light metal alloys and pure light metals. Smithells Light Metals Handbook.

[117] Stevanin T.M., Moir J.W.B., Read R.C. (2005). Nitric oxide detoxification systems enhance survival of *Neisseria meningitidis* in human macrophages and in nasopharyngeal mucosa. Infect. Immun..

[118] Fu X.T., Kim S.M. (2010). Agarase: Review of major sources, categories, purification method, enzyme characteristics and applications. Mar. Drugs.

[119] Sousa A.M.M., Rocha C.M.R., Goncalves M.P., Phillips G.O., Williams P.A. (2020). Agar. Handbook of Hydrocolloids.

[120] Yi-Rui Wu Y.-R., Zhang M.M., Zhong M., Hu Z. (2017). Synergistic enzymatic saccharification and fermentation of agar for biohydrogen production. Bioresour. Technol..

[121] Kim H.T., Lee S., Kim K.H., Choi I.-G. (2012). The complete enzymatic saccharification of agarose and its application to simultaneous saccharification and fermentation of agarose for ethanol production. Bioresour. Technol..

[122] Jagannathan S.V., Manemann E.M., Rowe S.E., Callender M.C., Soto W. (2021). Marine actinomycetes, new sources of biotechnological products. Mar. Drugs.

[123] Nussinovitch A. (1997). Hydrocolloid Applications: Gum Technology in the Food and Other Industries.

[124] Macian M.C., Ludwig W., Schleifer K.H., Pujalte M.J., Garay E. (2001). *Vibrio agarivorans* sp. nov., a novel agarolytic marine bacterium. Int. J. Syst. Evol. Microbiol..

[125] Doi H., Chinen A., Fukuda H., Usuda Y. (2016). *Vibrio algivorus* sp. nov., an alginate- and agarose-assimilating bacterium isolated from the gut flora of a turban shell marine snail. Int. J. Syst. Evol. Microbiol..

[126] Liu Y., Jin X., Wu C., Zhu X., Liu M., Call D.R., Zhao Z. (2020). Genome-wide identification and functional characterization of b-agarases in *Vibrio astriarenae* strain HN897. Front. Microbiol..

[127] Gomez-Gil B., Roque A., Rotllant R., Peinado L., Romalde J.L., Doce A., Cabanillas-Beltrán H., Chimetto L.A., Thompson F.L. (2011). *Photobacterium swingsii* sp. nov., isolated from marine organisms. Int. J. Syst. Evol. Microbiol..

[128] Lim E.-L., Siow R.-S., Rahim R.A., Ho C.-L. (2016). Global transcriptome analysis of *Gracilaria changii* (rhodophyta) in response to agarolytic enzyme and bacterium. Mar. Biotechnol..

[129] Armisen R., Galatas F., Phillips G.O., Williams P.A. (2009). Agar. Handbook of Hydrocolloids.

[130] Chi W.-J., Chang Y.-K., Hong S.-K. (2012). Agar degradation by microorganisms and agar-degrading enzymes. Appl. Microbiol. Biotechnol..

[131] Potin P., Amsler C.D. (2008). Oxidative burst and related responses in biotic interactions of algae. Algal Chemical Ecology.

[132] Cosse A., Leblanc C., Potin P. (2008). Dynamic defense of marine macroalgae against pathogens: From early activated to gene-regulated responses. Adv. Bot. Res..

[133] Cock J.M., Coelho S.M. (2011). Algal models in plant biology. J. Exp. Bot..

[134] Coelho S.M., Peters A.F., Müller D., Cock J.M. (2020). *Ectocarpus*: An evo-devo model for the brown algae. EvoDevo.

[135] Sørensen I., Rose J.K.C., Doyle J.F., Domozych D.S., Willats W.G.T. (2012). The charophycean green algae as model systems to study plant cell walls and other evolutionary adaptations that gave rise to land plants. Plant Signal. Behav..

[136] Knoll A.H. (2011). The multiple origins of complex multicellularity. Annu. Rev. Earth Planet. Sci..

[137] Tang L., Qiu L., Liu C., Du G., Mo Z., Tang X., Mao Y. (2019). Transcriptomic insights into innate immunity responding to red rot disease in red alga *Pyropia yezoensis*. Int. J. Mol. Sci..

[138] Thomas F., Cosse A., Le Panse S., Kloareg B., Potin P., Leblanc C. (2014). Kelps feature systemic defense responses: Insights into the evolution of innate immunity in multicellular eukaryotes. N. Phytol..

[139] Arnhold J. (2021). Heme peroxidases at unperturbed and inflamed mucous surfaces. Antioxidants.

[140] Weinberger F. (2007). Pathogen-induced defense and innate immunity in macroalgae. Biol. Bull..

[141] Kutty S.K., Ho K.K.K., Kumar N., Seabra A.B. (2017). Nitric oxide donors as antimicrobial agents. Nitric Oxide Donors: Novel Biomedical Applications and Perspectives.

[142] Kolbert Z., Lindermayr C., Loake G.J. (2021). The role of nitric oxide in plant biology: Current insights and future perspectives. J. Exp. Bot..

[143] Astier J., Rossi J., Chatelain P., Klinguer A., Besson-Bard A., Rosnoblet C., Jeandroz S., Nicolas-Francès V., Wendehenne D. (2021). Nitric oxide production and signalling in algae. J. Exp. Bot..

[144] Dit Frey N.F., Robatzek S. (2009). Trafficking vesicles: Pro or contra pathogens?. Curr. Opin. Plant Biol..

[145] Kindt T.J., Goldsby R.A., Osborne B.A. (2007). Cells and organs of the immune system. Kuby Immunology.

[146] Yutin N., Wolf M.Y., Wolf Y.I., Koonin E.V. (2009). The origins of phagocytosis and eukaryogenesis. Biol. Direct.

[147] Jones J.D.G., Dangl J.L. (2006). The plant immune system. Nature.

[148] Leary A.Y., Sanguankiattichai N., Duggan C., Tumtas Y., Pandey P., Segretin M.E., Linares J.S., Savage Z.D., Yow R., Bozkurt T.O. (2018). Modulation of plant autophagy during pathogen attack. J. Exp. Bot..

[149] Zeng H.-Y., Zheng P., Wang L.-Y., Bao H.-N., Sahu S.K., Yao N. (2019). Autophagy in plant immunity. Adv. Exp. Med. Biol..

[150] Paungfoo-Lonhienne C., Schmidt S., Webb R.I., Lonhienne T.G.A., De Bruijn F.J. (2013). Rhizophagy—A new dimension of plant–microbe interactions. Molecular Microbial Ecology of the Rhizosphere.

[151] White J.F., Torres M.S., Verma S.K., Elmore M.T., Kowalski K.P., Kingsley K.L., Singh A.K., Kumar A., Singh P.K. (2019). Evidence for widespread microbivory of endophytic bacteria in roots of vascular plants through oxidative degradation in root cell periplasmic spaces. PGPR Amelioration in Sustainable Agriculture: Food Security and Environmental Management.

[152] Murua P., Muller D.G., Etemadi M., Van West P., Gachon C.M.M. (2020). Host and pathogen autophagy are central to the inducible local defences and systemic response of the giant kelp *Macrocystis pyrifera* against the oomycete pathogen *Anisolpidium ectocarpii*. N. Phytol..

[153] Tamadoni Jahromi S.T., Barzkar N. (2018). Future direction in marine bacterial agarases for industrial applications. Appl. Microbiol. Biotechnol..

[154] Chen X., Fu X., Huang L., Xu J., Gao X. (2021). Agar oligosaccharides: A review of preparation, structures, bioactivities and application. Carbohydr. Polym..

[155] Davis D.J., Wang M., Sørensen I., Rose J.K.C., Domozych D.S., Drakakaki G. (2020). Callose deposition is essential for the completion of cytokinesis in the unicellular alga *Penium margaritaceum*. J. Cell Sci..

[156] Nedukha O.M. (2015). Callose: Localization, functions, and synthesis in plant cells. Cytol. Genet..

[157] Scherp P., Grotha R., Kutschera U. (2001). Occurrence and phylogenetic significance of cytokinesis-related callose in green algae, bryophytes, ferns and seed plants. Plant Cell Rep..

[158] Medzhitov R. (2021). The spectrum of inflammatory responses. Science.

[159] Burdett H., Bentham A.R., Williams S.J., Dodds P.N., Anderson P.A., Banfield M.J., Kobe B. (2019). The plant ‘‘resistosome’’: Structural insights into immune signaling. Cell Host Microbe.

[160] Coll N.S., Epple P., Dangl J.L. (2011). Programmed cell death in the plant immune system. Cell Death Differ..

[161] Gust A.A., Pruitt R., Nürnberger T. (2017). Sensing danger: Key to activating plant immunity. Trends Plant Sci..

[162] Klessig D.F., Tian M., Choi H.W. (2016). Multiple targets of salicylic acid and its derivatives in plants and animals. Front. Immunol..

[163] Lahaye M., Rochas C. (1991). Chemical structure and physico-chemical properties of agar. Hydrobiologia.

[164] Armisen R., Galatas F., McHugh D.J. (1987). Production, properties and uses of agar. Production and Utilization of Products from Commercial Seaweeds.

[165] Lee W.K., Lim Y.-Y., Leow A.T., Namasivayam P., Abdullah J.O., Ho C.L. (2017). Biosynthesis of agar in red seaweeds: A review. Carbohydr. Polym..

[166] Yoshida M.A., Tanabe T., Akiyoshi H., Kawamukai M. (2022). Gut microbiota analysis of Blenniidae fishes including an algae-eating fish and clear boundary formation among isolated *Vibrio* strains. Sci. Rep..

[167] Anaya-Rosasa R.E., Rivas-Vega M.E., Miranda-Baeza A., Piña-Valdez P., Nieves-Soto M. (2019). Effects of a co-culture of marine algae and shrimp *(Litopenaeus vannamei*) on the growth, survival and immune response of shrimp infected with *Vibrio parahaemolyticus* and white spot virus (WSSV). Fish Shellfish. Immunol..

[168] Koedooder C., Van Geersdaele R., Gueneugues A., Bouget F.-Y., Obernosterer I., Blain S. (2020). The interplay between iron limitation, light and carbon in the proteorhodopsin-containing *Photobacterium angustum* S14. FEMS Microbiol. Ecol..

[169] Wang Z., O’Shaughnessy T.J., Soto C.M., Rahbar A.M., Robertson K.L., Lebedev N., Vora G.J. (2012). Function and regulation of *Vibrio campbellii* proteorhodopsin: Acquired phototrophy in a classical organoheterotroph. PLoS ONE.

[170] Wang L., Chen Y., Huang H., Huang Z., Chen H., Shao Z. (2015). Isolation and identification of *Vibrio campbellii* as a bacterial pathogen for luminous vibriosis of *Litopenaeus vannamei*. Aquac. Res..

[171] Amaral G.R., Silva B.S.D., Santos E.O., Dias G.M., Lopes R.M., Edwards R.A., Thompson C.C., Thompson F.L. (2012). Genome sequence of the bacterioplanktonic, mixotrophic *Vibrio campbellii* strain PEL22A, isolated in the Abrolhos Bank. J. Bacteriol..

[172] Gomez-Consarnau L., Akram N., Lindell K., Pedersen A., Neutze R., Milton D.L., Gonzalez J.M., Pinhassi J. (2010). Proteorhodopsin phototrophy promotes survival of marine bacteria during starvation. PLoS Biol..

[173] Palovaara J., Akram N., Baltar F., Bunsea C., Forsberga J., Pedrós-Aliób C., González J.M., Pinhassi J. (2014). Stimulation of growth by proteorhodopsin phototrophy involves regulation of central metabolic pathways in marine planktonic bacteria. Proc. Natl. Acad. Sci. USA.

[174] Martinez A., Bradley A.S., Waldbauer J.R., Summons R.E., DeLong E.F. (2007). Proteorhodopsin photosystem gene expression enables photophosphorylation in a heterologous host. Proc. Natl. Acad. Sci. USA.

[175] Steindler L., Schwalbach M.S., Smith D.P., Chan F., Giovannoni S.J. (2011). Energy starved *Candidatus* Pelagibacter ubique substitutes light-mediated atp production for endogenous carbon respiration. PLoS ONE.

[176] Van de Water J.A.J.M., Allemand D., Ferrier-Pagès C. (2018). Host-microbe interactions in octocoral holobionts-recent advances and perspectives. Microbiome.

[177] Basu P., Sandhu N., Bhatt A., Singh A., Balhana R., Gobe I., Crowhurst N.A., Mendum T.A., Gao L., Ward J.L. (2018). The anaplerotic node is essential for the intracellular survival of *Mycobacterium tuberculosis*. J. Biol. Chem..

[178] Kappelmann J., Wiechert W., Noack S. (2016). Cutting the gordian knot: Identifiability of anaplerotic reactions in *Corynebacterium glutamicum* by means of 13 c-metabolic flux analysis. Biotechnol. Eng..

[179] Brissette J.L., Russel M., Weiner L., Model P. (1990). Phage shock protein, a stress protein of *Escherichia coli*. Proc. Natl. Acad. Sci. USA.

[180] Huvet H., Toni T., Sheng X., Thorne T., Jovanovic G., Engl C., Buck M., Pinney J.W., Stumpf M.P.H. (2011). The evolution of the phage shock protein response system: Interplay between protein function, genomic organization, and system function. Mol. Biol. Evol..

[181] Flores-Kim J., Darwin A.J. (2016). The phage shock protein response. Annu. Rev. Microbiol..

[182] Ravi J., Anantharaman V., Aravind L., Gennaro M.L. (2018). Variations on a theme: Evolution of the phage-shock-protein system in actinobacteria. Antonie Van Leeuwenhoek.

[183] Huvet M., Toni T., Tan H., Jovanovic G., Engl C., Buck M., Stump M.P.H. (2009). Model-based evolutionary analysis: The natural history of phage-shock stress response. Biochem. Soc. Trans..

[184] Manganelli R., Gennaro M.L. (2017). Protecting from envelope stress: Variations on the phage-shock-protein theme. Trends Microbiol..

[185] Tsai W.-C., Kuo T.-Y., Lin C.-Y., Lin J.-C., Chen W.-J. (2014). *Photobacterium damselae* subsp. *piscicida* responds to antimicrobial peptides through phage-shock-protein A (pspA)-related extracytoplasmic stress response system. J. Appl. Microbiol..

[186] DeAngelis C.M., Nag D., Withey J.H., Matson J.S. (2019). Characterization of the *Vibrio cholerae* phage shock protein response. J. Bacteriol..

[187] Kimura M. (1983). Overdevelopment of the synthetic theory and the proposal of the neutral theory. The Neutral Theory of Molecular Evolution.

[188] Lynch M. (2010). Evolution of the mutation rate. Trends Genet..

[189] Wielgoss S., Barrick J.E., Tenaillon O., Wiser M.J., Dittmar W.J., Cruveiller S., Chane-Woon-Ming B., Médigue C., Lenski R.E., Schneider D. (2013). Mutation rate dynamics in a bacterial population reflect tension between adaptation and genetic load. Proc. Natl. Acad. Sci. USA.

[190] Blountsas Z.D., Lenski R.E., Losos J.B. (2018). Contingency and determinism in evolution: Replaying life’s tape. Science.

[191] Chavez-Dozal A., Soto W., Nishiguchi M.K. (2021). Identification of a transcriptomic network underlying the wrinkly and smooth phenotypes of *Vibrio fischeri*. J. Bacteriol..

[192] Hoffmann R., Slattery J.S., Kruta I., Linzmeier B.J., Lemanis R.E., Mironenko A., Goolaerts S., De Baets K., Peterman D.J., Klug C. (2021). Recent advances in heteromorph ammonoid palaeobiology. Biol. Rev..

[193] Gould S.J. (1989). Replaying life’s tape: The crucial experiment. Wonderful Life: The Burgess Shale and the Nature of History.

[194] Kapodistrias A., Katsiampoura G., Skordoulis C. (2022). Emergence and contingency in modern scientific theories. Adv. Hist. Stud..

[195] Kauffman S.A. (1993). Dynamical systems and their attractors. The Origins of Order: Self-Organization and Selection in Evolution.

[196] Gleick J. (1987). The butterfly effect. Chaos: Making a New Science.

[197] Travisano M., Mongold J.A., Bennett A.F., Lenski R.E. (1995). Experimental tests of the roles of adaptation, chance, and history in evolution. Science.

[198] Bruger E.L., Snyder D.J., Cooper V.S., Waters C.M. (2020). Quorum sensing provides a molecular mechanism for evolution to tune and maintain investment in cooperation. ISME J..

[199] Soto W., Punke E.B., Nishiguchi M.K. (2012). Evolutionary perspectives in a mutualism of sepiolid squid and bioluminescent bacteria: Combined usage of microbial experimental evolution and temporal population genetics. Evolution.

